# Green Multi-Platform Solution for the Quantification of Levodopa Enantiomeric Excess in Solid-State Mixtures for Pharmacological Formulations

**DOI:** 10.3390/molecules26164944

**Published:** 2021-08-15

**Authors:** Alessandra Biancolillo, Stefano Battistoni, Regina Presutto, Federico Marini

**Affiliations:** 1 Department of Physical and Chemical Sciences, University of L’Aquila, Via Vetoio, 67100 L’Aquila, Italy; 2 Department of Chemistry, University of Rome “La Sapienza”, Piazzale Aldo Moro 5, 00185 Rome, Italy; stefano.battistoni@aol.com (S.B.); reginapresutto@hotmail.it (R.P.)

**Keywords:** l-3,4-dihydroxyphenylalanine (l-DOPA), enantiomeric excess, near infrared (NIR) spectroscopy, mid-infrared (MIR) spectroscopy, multi-block data analysis, chemometrics, sequential and orthogonalized partial least squares (SO-PLS), sequential and orthogonalized covariance selection (SO-CovSel)

## Abstract

The aim of the present work was to develop a green multi-platform methodology for the quantification of l-DOPA in solid-state mixtures by means of MIR and NIR spectroscopy. In order to achieve this goal, 33 mixtures of racemic and pure l-DOPA were prepared and analyzed. Once spectra were collected, partial least squares (PLS) was exploited to individually model the two different data blocks. Additionally, three different multi-block approaches (mid-level data fusion, sequential and orthogonalized partial least squares, and sequential and orthogonalized covariance selection) were used in order to simultaneously handle data from the different platforms. The outcome of the chemometric analysis highlighted the quantification of the enantiomeric excess of l-DOPA in enantiomeric mixtures in the solid state, which was possible by coupling NIR and PLS, and, to a lesser extent, by using MIR. The multi-platform approach provided a higher accuracy than the individual block analysis, indicating that the association of MIR and NIR spectral data, especially by means of SO-PLS, represents a valid solution for the quantification of the l-DOPA excess in enantiomeric mixtures.

## 1. Introduction

3,4-dihydroxyphenylalanine, an amino-acid better known as DOPA, is a chiral active pharmaceutical ingredient (API) consisting of two enantiomers, l-DOPA (levodopa) and d-DOPA, particularly used to treat medical conditions resulting in dopamine deficiency (e.g., Parkinson’s disease). Of the two enantiomers, d-DOPA is inactive, whereas l-DOPA is able to cross the blood-brain barrier (bbb) and can therefore provide the required pharmaceutical effect. l-dopa is a prodrug—in its own form it is inactive—however, once it crosses the blood–brain barrier (bbb) through a system of transporters, it can be metabolized into dopamine by dopa-decarboxylase.

In general, racemic DOPA is orally administered in combination with inhibitors of peripheral decarboxylases. It is therapeutically not active but is useful for increasing the effectiveness of treatment and decreasing the risk of side effects [1].

Since the 1980s, awareness of the different biochemical and pharmacological properties of diverse enantiomers led many national and supranational organizations to promote the pharmaceutical industry to develop and commercialize chiral drugs in enantiopure forms rather than as racemic mixtures [2]. Nevertheless, it is not always possible to perform enantioselective syntheses; more often, racemic products are synthesized and then enantiomeric excesses in the formulation are determined.

The most natural way of assessing the enantiomeric excess in formulations is by means of polarimetric analysis, exploiting the rotatory power of the API [3]. This approach is the one suggested by the *European Pharmacopoeia*; nevertheless, the application of this technique is not the most suitable solution for small concentrations of the enantiomer of interest.

Consequently, the enantiomeric excess in pharmaceutical formulations is generally determined by means of chiral chromatographic techniques [4]. For instance, as described by Doležalová and Tkaczyková [5], l-DOPA can be quantified by high-performance liquid chromatography (HPLC) equipped with an ordinary C18 column, but using a chiral mobile phase containing *N*,*N*-dimethyl-l-phenylalanine and Cu(II) acetate (achieving a detection limit of 0.04% for the d-enantiomer in l-DOPA). The same authors have demonstrated that this goal can be achieved using a teicoplanin column and an ethanol–water (65:35, *v*/*v*) mobile phase.

An alternative solution for the quantification of l-DOPA in formulations based on capillary electrophoresis has been proposed by Blanco and Valverde [6]. In their work, the separation of the enantiomers is achieved by including a chiral selector ((+)-(18-crown-6)-2,3,11,12-tetracarboxylic acid) in the background electrolyte. In this way, the authors achieved a relative limit of detection for d-DOPA (contained in l-DOPA) of 0.1%.

In addition to this routine solution, in recent years, the possibility of quantifying enantiomeric excess in the solid state by means of infrared spectroscopic techniques has emerged. This is possible because in the crystalline phase the enantiomers and the corresponding racemic compound may possess different chemical-physical properties depending on the relative affinity of the two enantiomeric forms. It follows that it is possible to quantify the enantiomeric excess of the active ingredients in solid formulations [7]. This is particularly sound because it indicates the possibility of determining enantiomeric excess in solid samples by means of green, fast, and relatively cheap, procedures. In particular, the application of near or mid-infrared spectroscopy (NIR/MIR) in this context is of interest because, besides being a rapid and green approach, it is commonly used for online monitoring in pharmaceutical industries. In the literature, the application of these spectroscopic techniques with the aim of quantifying compounds in mixtures without the need of a reference procedure has been widely discussed in different contexts; for instance, for the quantification of active ingredients in semi-solid pharmaceutical formulations [8,9], or, in food analysis, for the estimation of adulterants or quantification of compounds [10,11,12,13].

The application of MIR and NIR in this regard is strictly related to their combination with chemometric methods that allows extracting information from the spectra and quantifying the different enantiomeric forms in the mixtures. Despite the number of advantages that a green and fast procedure for the enantiomeric excess quantification has, in the literature, the possibility of using NIR or MIR for the quantification of enantiomers in mixture has been described only for a reduced number of compounds of pharmacological interest. In particular, this has been accomplished by means of MIR for mandelic acid and ketoprofen [14], and by NIR for ibuprofen, epinephrine [15], and tartaric acid [16]. In the former case, mandelic acid and ketoprofen were quantified by means of a chemometric regression strategy based on partial least squares (PLS) [17,18] and by exploiting a multivariate curve resolution (MCR). Eventually, Marini et al. concluded that the most suitable solution was represented by backward interval PLS coupled with genetic algorithms (bi-PLS-GA). Concerning the NIR-based quantification of APIs, in the case of tartaric acid, the coupling of this technique with a regression model led to a limit of quantification (LOQ) of 0.5%, and a relatively low error (between 2.5% and 5%). Conversely, the estimation of the enantiomeric excess in ibuprofen and epinephrine was based on a more elaborate chemometric strategy; enantiomers were quantified by PLS and then a model was interpreted by variable importance in projection (VIP) analysis. This strategy allowed achieving accurate predictions (root mean square error in prediction (RMSEP) <2% for both compounds).

Considering this evidence, the aim of the present work is to develop a green multi-platform methodology for the quantification of l-DOPA in enantiomeric mixtures by means of MIR and NIR spectroscopy. In order to achieve this goal, different regression strategies were chosen: partial least square (PLS) [17,18], mid-level data fusion on PLS’ scores [19], sequential and orthogonalized partial least squares (SO-PLS) [20,21], and sequential and orthogonalized covariance selection (SO-CovSel). Of these, the former is probably the most widely applied chemometric regression method; in its basic formulation, it can handle one data block individually, but it was chosen because it is particularly suitable for quantification of analytes in mixtures [22], and it is commonly applied for API quantification [23,24,25]. Conversely, multi-block approaches allow the simultaneous modeling of both data matrices. Mid-level data fusion is one of the most natural extensions of PLS in the multi-block field, and it has demonstrated to be a performant solution in similar contexts [26,27,28,29]. Nevertheless, results can be affected by possible redundancies present in data. Consequently, SO-PLS and SO-CovSel, which were conceived to overcome these drawbacks, were tested. Compared to other data fusion approaches, these have the advantage of removing redundant information among the predictor blocks; due to the nature of the multi-set data set, this represents a crucial characteristic, which makes this approach particularly advisable for the aim of the present work [30,31,32,33,34,35,36,37].

## 2. Results and Discussion

Prior to chemometric analysis, NIR and MIR signals (collected in reflectance (R) and transmittance (T) mode, respectively) were transformed into pseudo-absorbance ($A_{pseudo}$ = log(1/R)) and absorbance (*A* = log(1/T), respectively). Thereafter, spectral replicates were averaged, obtaining two data blocks, *X_MIR_* and *X_NIR_*, of dimensions 33 × 3601 and 33 × 3112, respectively.

**Figure 1:**
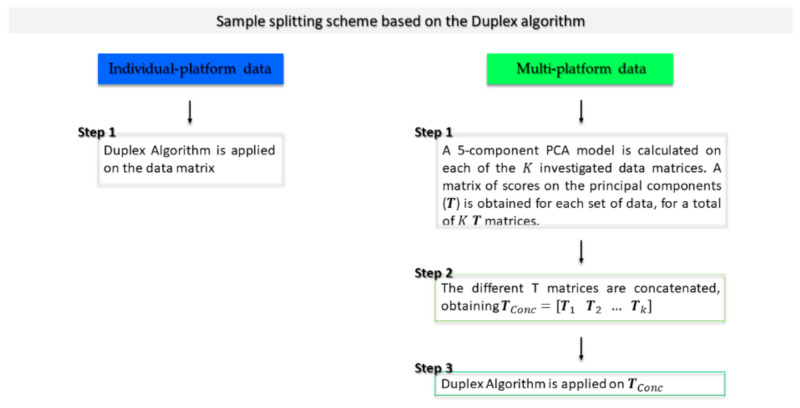
Scheme of the duplex-based splitting strategy.

In order to perform external validation of the models, a reorganization of data into a training and a test set was necessary. In a situation in which the instrumental analysis would be conducted by means of an individual technique, this could be achieved by direct application of a resampling algorithm (such as Duplex [38]) but this was not possible with a multi-platform data set. The application of the Duplex algorithm on the individual data blocks would not simultaneously allow consideration of the variability present in the two matrices. In order to overcome this issue, a solution based on principal component analysis (PCA) [39,40] proposed by Firmani et al. [41], and schematized in Figure 1, was applied. Consequently, a PCA model was calculated on each set of mean-centered data. The samples’ scores, along the first 5 principal components of each model (arranged in the matrices $T_{MIR\;}$ and $T_{NIR\;}$), were extracted and concatenated row-wise, obtaining the row-augmented score matrix $T_{Conc}$ ($T_{Conc}=[T_{MIR\;}\;T_{NIR}$]). Eventually, the Duplex algorithm was applied on $T_{Conc}$ and signals divided into a training and a test set of 23 and 10 samples, respectively.

As mentioned above, the aim of the present study is the development of a multi-block method for the quantification of l-DOPA excess in DOPA mixtures, possibly containing both enantiomers. The sequential analysis of the multi-block dataset was performed by means of SO-PLS. Nevertheless, PLS-based individual block analysis was performed for comparison. The details associated with the model building and the outcomes of these analyses are reported below in the related sub-sections.

**Figure 2:**
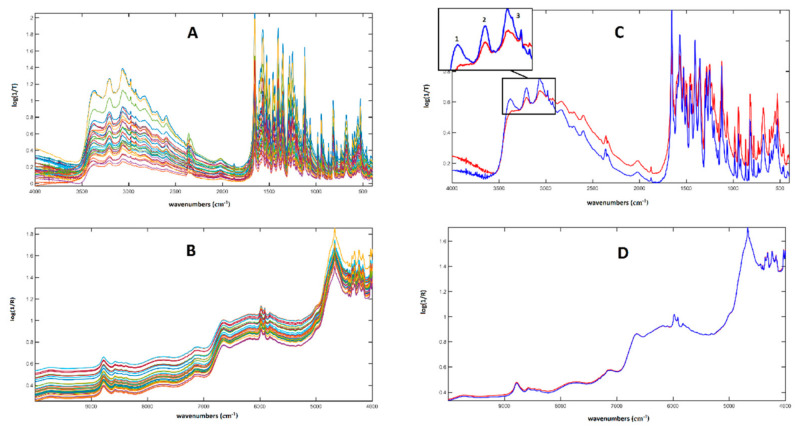
Graphical representation of the spectroscopic data collected for the present study. (**A**) MIR spectra of the 33 enantiomeric mixtures in the solid state (KBr pellet); (**B**) NIR spectra of the 33 enantiomeric mixtures in the solid state (integrating sphere); (**C**) average MIR spectra for Pure l-DOPA (red) and racemic DOPA (blue); (**D**) average NIR spectra for Pure L-DOPA (red) and racemic DOPA (blue).

A graphical representation of the MIR and NIR spectra collected on all the investigated mixtures is reported in Figure 2A,B, whereas the MIR and NIR average spectra for pure l-DOPA and for the racemic mixtures are displayed in Figure 2C,D, respectively. From Figure 2C, the discrepancy between the pure l- and the racemic DOPA is evident. In particular, the three peaks at 3370 cm^−1^, 3206 cm^−1^, and 3070 cm^−1^ (labeled as 1, 2, and 3 in Figure 2C) were associated with asymmetric and symmetric NH stretching and arylic CH stretching, respectively, and were less intense in l-DOPA than in *rac*-DOPA; in contrast, the peaks ascribable to the CH vibrations of aliphatic CH bonds, i.e., those in the spectral area between 2845 cm^−1^ and 3000 cm^−1^, were more intense in the spectrum of L-DOPA [42]. In the area between 3500 cm^−1^ and 2200 cm^−1^, a strong contribution of the OH stretching signal, broadened by hydrogen bonding, and imputable to the carboxylic function and water content, was also observed. The pure and the racemic mixture did not present sensible differences in the NIR spectrum; the two average signals almost completely overlapped.

### 2.1. MIR and NIR Single Block Analysis

MIR and NIR spectra were individually elaborated to quantify the l-DOPA in mixtures by means of PLS. Different data preprocessing strategies were tested, and as many regression models as the number of pretreatments used were developed. Then, regardless of the platform used to collect signals (NIR or MIR), the optimal data preprocessing and the number of latent variables (LVs) to be extracted were defined by inspection of the root mean square error (RMSECV) in a (8-fold) cross-validation procedure. Among the different models, the one leading to the lowest RMSECV was chosen as the optimal one and applied on the test set.

The tested pretreatments were: first derivative (19 points window, second order polynomial), second derivative (19 points window, third order polynomial), standard normal variate (SNV), and their combinations. Signals were mean-centered (MC) prior to the creation of the regression models; the RMSECV obtained building the different PLS models are reported in Table 1, together with the other model parameters.

Concerning the models built on MIR data prior to analysis, the profiles were cut to 3541 cm^−1^ (3142 data points) since the higher wavenumbers were mostly baseline. The optimal one resulted in Model Va (i.e., the model calculated on data preprocessed by SNV and the first derivative), which led to the lowest RMSECV (18.1). Its application for the quantification of l-DOPA in the test samples led to a root mean square error in prediction (RMSEP) of 10.9.

**Figure 3:**
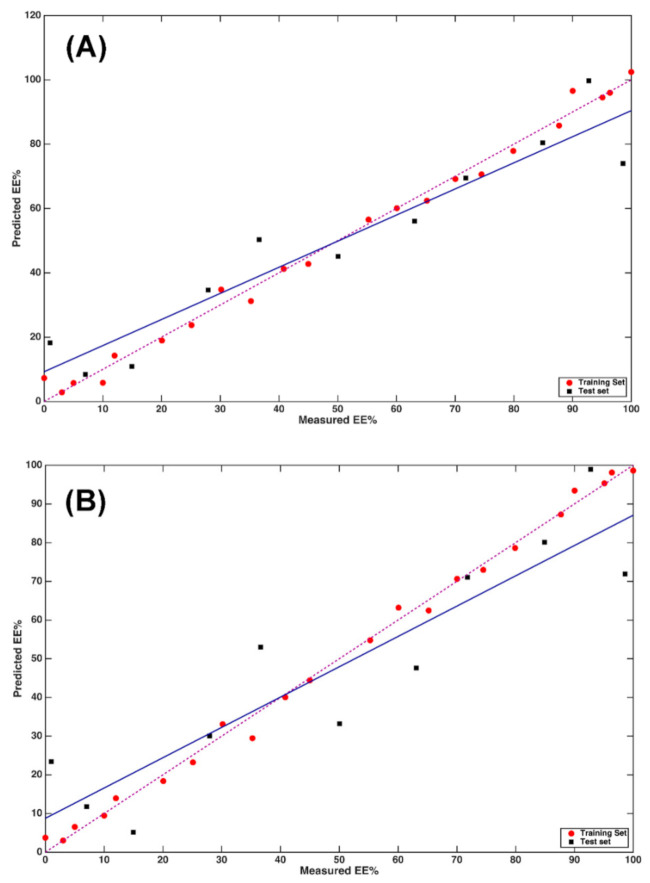
PLS analysis on MIR data: plot of predicted vs. measured Y (EE%). (**A**) PLS model calculated on MIR data; (**B**) PLS model calculated on MIR data after variable reduction, based on the values of the VIP scores. Red circles and black squares indicate training and test samples, respectively. The actual fit based on the predictions on the test samples is represented by the blue solid line, whereas the dashed purple line corresponds to the ideal fit.

The fit associated with this regression model is displayed in Figure 3A. In the plot, red and black symbols represent training and test samples, respectively; the solid blue line depicts the fit of the predictions on the test set, whereas the dashed purple line represents the ideal fit. The suitability of the closeness of the two lines of the model in predicting the enantiomeric excess on the basis of MIR data is apparent, and this is confirmed by the values of the R^2^_p_ (0.89) and bias_p_ (0.1).

**Figure 4:**
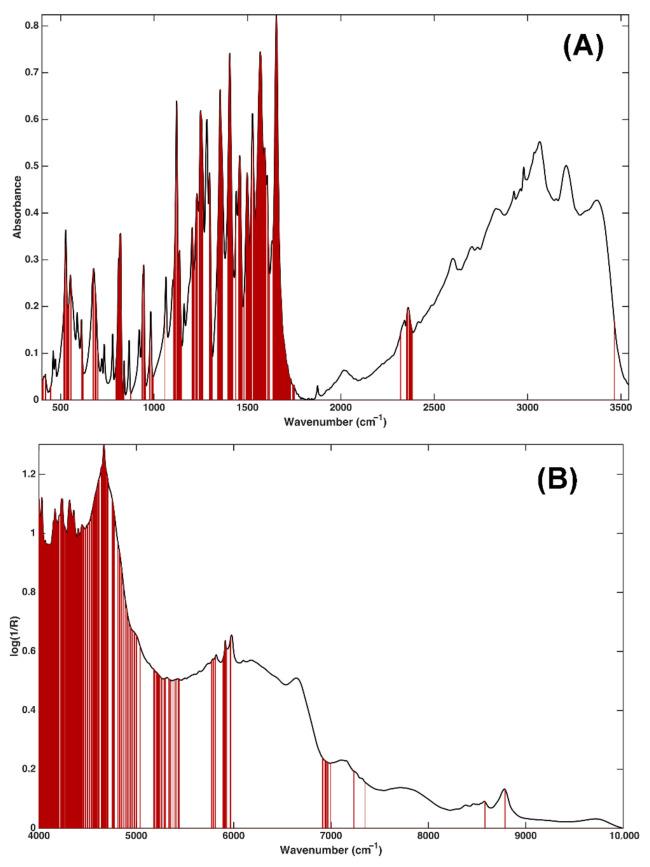
PLS analysis: identification of the variables contributing the most to the calibration models based on the values of the VIP indices. Dark red vertical bars represent the predictors identified as significantly contributing to the model built on: (**A**) MIR; and (**B**) NIR spectra.

Eventually, in order to understand which spectral variables contributed the most to the quantification of l-DOPA, VIP analysis [43,44] was performed. A total of 634 variables (over 3142) presented a VIP index of higher than 1, indicating their relevance in the definition of the regression model. A graphical representation of these features is shown in Figure 4A. In the plots, the black solid line represents the average spectrum, whereas the variables presenting a VIP index of >1 are highlighted in dark red.

The inspection of the outcome of VIP analysis confirms certain hypotheses drawn at the beginning. In particular, the investigation of Figure 4A indicates only several variables in the range 3500–2100 cm^−1^ are relevant; however, the entire fingerprint region contributes to the quantification of l-DOPA. Among the variables presenting VIP index of >1, it is possible to recognize the typical vibrations of amino acids, e.g., deformation of the NH bonds between 1560 and 1650 cm^−1^ partially superimposed on the C=O stretching of the carboxyl group. It is also possible to notice the phenolic CO stretching (approximately at 1120 cm^−1^) and the out-of-plane deformations of the three aromatic CH bonds (between 810 and 830 cm^−1^) typical of aromatic compounds, which have been ranked as the most relevant by VIP analysis.

These observations are also confirmed by the most influencing NIR variables (Figure 4B). The features presenting a VIP index of higher than 1 are those ascribable to the combination of N-H vibrations (symmetric or asymmetric stretching with NH_2_ scissoring or rocking), giving rise to the peaks between 4500 and 5020 cm^−1^ and to the combination of the C=O and OH stretching modes at about 5290 cm^−1^ in which the second overtone of C=O stretching also falls. Lastly, a significant contribution of the first overtone of the aromatic C–H stretching at about 5950 cm^−1^ is also observed.

In order to evaluate whether to only consider that the most relevant variables for the calibration could improve predictive ability, a second PLS model was calculated, including the 634 wavenumbers identified based on their VIP scores. The optimal complexity of the model was found to be 11 LVs, leading to an RMSECV of 14.2. When the model was applied to the 11 test samples, an RMSEP of 14.1 was obtained; bias_p_ and R^2^_p_ were 2.1 and 0.82, respectively.

The results are also graphically displayed as predicted vs. observed EE% values in Figure 3B.

The pattern of the predicted points on the plot confirms what was already indicated by the calculated figures of merit, i.e., that including only the predictors with VIP scores higher than one does not improve the predictive ability of the model built on MIR data, but leads to worse results.

**Figure 5:**
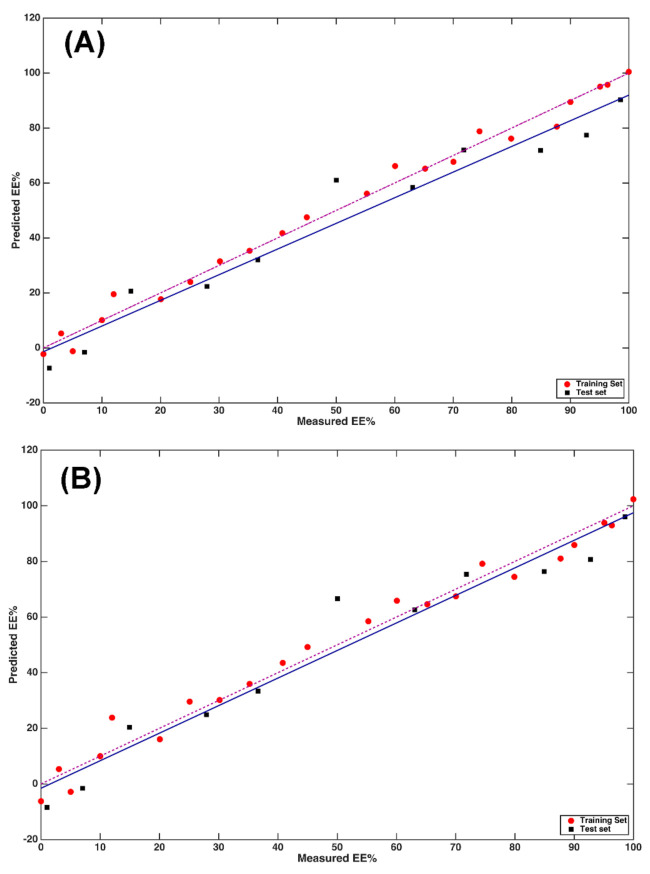
PLS analysis on NIR data: plot of predicted vs. measured Y (EE%). (**A**) PLS model calculated on NIR data; (**B**) PLS model calculated on NIR data after variable reduction based on the values of the VIP scores. Red circles and black squares indicate training and test samples, respectively. The actual fit based on the predictions on the test samples is represented by the blue solid line, whereas the dashed purple line corresponds to the ideal fit.

The same model-building pipeline was followed in the case of NIR data. In particular, when looking at the RMSECV values obtained on the differently pretreated spectra (Table 2), it can be observed how results are generally better than those obtained on the NIR spectra. In the case of NIR profiles, the optimal model was Model VIb, i.e., the model built on data preprocessed by SNV and the second derivative, which led to an RMSECV of 10.8, and once applied to the test set, to an RMSEP of 8.8 with bias_p_ = 4.7 and R^2^_p_ = 0.93. The fit associated to this model is displayed in Figure 5A, where it is evident that the majority of the test data are predicted with satisfactory accuracy and the relatively high value of bias being ascribable to a several samples.

In this case, inspection of the VIP indices allowed interpretation of the optimal model in terms of the spectral regions contributing the most to its definition. In particular, 536 variables (over 3112) presented a VIP score of higher than one. The variables are graphically displayed in Figure 4B. It is apparent from the figure how certain considerations reported above find a partial confirmation by inspecting the most relevant NIR variables. The peak at (approximately) 5220 cm^−1^, related to carboxylic compounds, and the second overtone of C=O at 5900 cm^−1^ were ranked as highly contributing to the regression model. In addition to these, other few variables ascribable to humidity (between 4000 and 5000 cm^−1^) present VIP indices of >1, suggesting that this information may contribute to the quantification of l-DOPA in mixtures.

As in the case of MIR data, a second PLS model was built by including only the wavenumbers that were identified as relevant based on their VIP score. The resulting model had an optimal complexity of 6 LVs and led to an RMSECV of 9.7. When applied to the test samples, it resulted in a good predictive ability since the RMSEP was 8.1 and the R^2^_p_ = 0.94 with bias_p_ = 2.0. These results can be graphically appreciated as shown in Figure 5B, where the predicted vs. observed EE% values are displayed for both training and test samples.

In the case of NIR, different from what was observed for the MIR data and restricting the analysis to the most relevant variables only, allowed improvement to the predictive ability of the model, including its interpretability. This is also apparent from the closeness between the lines of the actual and the ideal fit.

The results obtained on the individual blocks indicate that the use of NIR spectra to quantify the enantiomeric excess of l-DOPA in the solid state led to significantly better results than obtained with MIR. This observation may be linked to the higher noise of the MIR measurements, or to its higher sensitivity to the presence of humidity.

### 2.2. Multi-Block Analysis

In the second phase of the study, to verify whether their combination could lead to more accurate predictions, multi-block approaches were used to integrate NIR and MIR data into a joint model.

**Figure 6:**
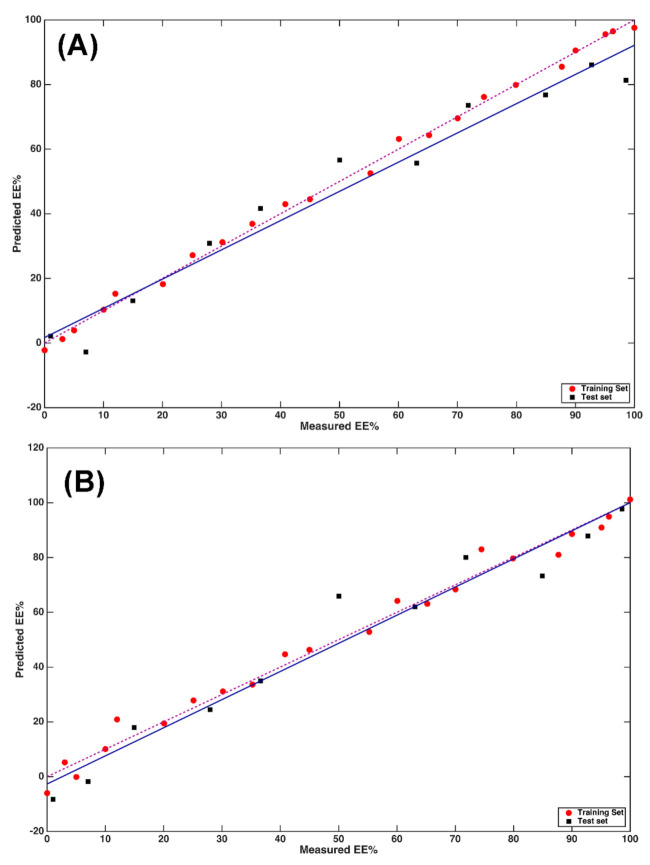
Calibration results after fusion of MIR and NIR data: plot of predicted vs. measured Y (EE%). (**A**) Multi-block PLS model calculated on MIR and NIR data; (**B**) SO-PLS model calculated on MIR and NIR data. Red circles and black squares indicate training and test samples, respectively. The actual fit based on the predictions on the test samples is represented by the blue solid line, whereas the dashed purple line corresponds to the ideal fit.

At first, a mid-level data fusion strategy was followed by fusing the scores of the PLS models built on the individual data sets after VIP-based variable selection (i.e., the models displayed in Figure 3B and Figure 5B, respectively), and the concatenated matrices obtained with this method on the training and test data were used for model building and model validation, respectively. PLS was then applied to the concatenated score matrix and the optimal complexity was chosen again as the one leading to the lowest RMSECV in 8-fold cross-validation. It resulted to be 5 LVs, corresponding to an RMSECV of 10.2. When this model was applied to the test set, it resulted in RMSEP = 7.6, R^2^_p_ = 0.95, and bias_p_ = 3.1. The results are graphically displayed in Figure 6A.

By inspecting the figure, it can be observed how predictions are generally better than those obtained by all other models as summarized by the RMSEP values and, at the same time, that the slightly higher bias with respect to the model built only on NIR data should be ascribed to two samples that are predicted to be worse. 

To fuse the information from the two spectral platforms, another multi-block approach, namely, sequential and orthogonalized PLS (SO-PLS), was used. In order to build SO-PLS models, data were preprocessed according to the outcomes of the analyses on the individual blocks. Consequently, MIR data were processed by SNV and the first derivative, while NIR spectra were processed by SNV and the second derivative. In both cases, only the variables identified as relevant based on the values of the VIP indices were retained (using the embedded strategy described in [45]). All blocks were further mean-centered prior to the creation of the models.

Similar to PLS analysis, the optimal combination of LVs to be extracted by the individual blocks was defined by inspecting the RMSECV obtained by calculating the SO-PLS models on the training signals.

As discussed in [21], when building a SO-PLS model, the order of the input blocks should not affect predictions. As a further confirmation, two different SO-PLS models were created, testing the two possible orders of the blocks (NIR as first input block and MIR as the second, and vice versa). As expected, from the prediction point of view, no relevant differences were noticed; consequently, only the results obtained modeling NIR as the first input block and MIR as second are discussed below.

All the possible combinations of LVs (under a maximum value of 12 per each block) were tested and investigated by means of a Måge plot [21]. The model obtained by extracting 9 LVs from the NIR block, and 2 from the MIR led to an RMSECV of 10.4 with a R2cv of 0.90. The application of this model to the test set led to an RMSEP of 7.8, R^2^_p_ = 0.95, and bias_p_ = 1.3. The results are graphically displayed in Figure 6B.

The model has a comparable RMSEP and R^2^_p_ with respect to the one build based on the mid-level data fusion approach, but presents a significantly lower bias, as also indicated by the closeness between the lines corresponding to the actual and ideal fit on the plot, thus suggesting that the integration of the spectral information through SO-PLS is the best approach for building a calibration model for the prediction of the enantiomeric excess of l-DOPA in the solid phase.

Lastly, a second multi-block sequential regression method—sequential and orthogonalized covariance selection (SO-CovSel) [46], which couples a parsimonious variable selection strategy with multi-block calibration—was used. In this case, the model selection stage required the identification of the optimal number of experimental variables to be retained from each block of data, which was performed based on the minimum RMSECV and through the construction of a Måge plot, similar to the one already described for SO-PLS. The optimal model required the inclusion of only five experimental variables (wavenumbers), four from the NIR block, and 1 from the MIR. When applied to the test set, it provided good results, considering that it was built on only five predictors (RMSEP of 12.0, R^2^_p_ = 0.87, and bias_p_ = 1.7), but was significantly worse than those of SO-PLS.

## 3. Materials and Methods

### 3.1. Sample Preparation

**Figure 7:**
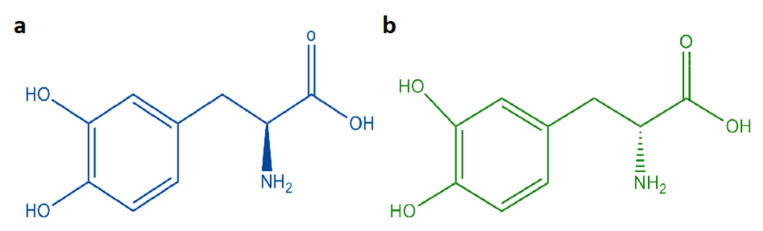
(**a**) L-DOPA, (**b**) D-DOPA.

A total of 33 mixtures of racemic and pure L-DOPA were prepared using certified standards purchased at Sigma-Aldrich (St. Louis, MO, USA). The chemical structures of the two compounds are shown in Figure 7. Details about the masses of the two constituents of the mixtures and the consequent percentage of enantiomeric excess are shown in Table 2. All the mass measurements were performed using a Gibertini E50S analytical scale (Novate Milanese, Italy).

### 3.2. Spectroscopic Analysis

NIR signals were collected using a Nicolet 6700 FT-NIR (Thermo Scientific Inc., Madison, WI, USA) equipped with an integrating sphere (Thermo Scientific Inc., Madison, WI, USA), which allowed a direct analysis of the mixtures, avoiding any physical pretreatment of the samples.

An aliquot of each mixture was introduced into a glass vial (designed with the same dimensions of the integrating sphere’s window) and NIR spectra in the 4000–10000 cm^−1^ range were recorded (nominal resolution: 4 cm^−1^) in reflectance mode. Three analytical replicates were collected for each mixture, and then NIR signals were exported by means of the OMNIC software (Thermo Scientific Inc., Madison, WI, USA).

Mid-infrared spectra were recorded by means of a PerkinElmer 1600 Series FT-IR spectrometer (PerkinElmer, San José, CA, USA), furnished with a Globar source and a DTGS detector, inspecting the spectral range between 400 and 4000cm^−1^ (nominal resolution: 4cm^−1^). Sample preparation consisted of incorporating an aliquot of each mixture with KBr, gently homogenizing all the analytes in an agate mortar, and setting the resulting powder in a tablet press. Two analytical replicates of each sample were investigated for a total of 66 MIR spectra collected. Data were exported through the software Spectrav 1.50 (PerkinElmer, San José, CA, USA).

Regardless of the platform used for data collection, the subsequent chemometric analysis was performed by means of in-house written functions running in Matlab (v. 8.6, release 2015b; The Mathworks, Natick, MA, USA).

### 3.3. Multivariate Regression Methods

#### 3.3.1. Partial Least Squares (PLS)

Partial least squares [17,18,22] is a well-known and widely applied regression approach, which allows modeling of the relationship between a dependent set of variables (a Y response) and a set of predictors (X matrix). PLS is a very efficient well-performant regression tool, and it is particularly useful for the quantification of analytes in mixtures based on spectroscopic measurements due to its ability to cope with many correlated variables; for this reason, it is widely used in pharmaceutical analysis [47].

For a single response case (y), as the one in the present work, the algorithm iteratively extracts X-scores (t) presenting the highest covariance with y. Essentially, for the first component ($t_{1}$), this corresponds to finding the direction $r_{1}$ such that the covariance between $t_{1}$ and y is maximum:

(1) $$\underset{r_{1}}{\mathrm{argmax}}\left[Cov\left(t_{1},y\right)\right]$$

Further components can be extracted according to the same criterion, with the additional constraint of orthogonality with respect to previous scores. Once the desired number of components (*F*, usually selected on the basis of cross-validation) is calculated, the response is regressed onto the scores ($T=\left[t_{1}\;t_{2}\ldots \;t_{F}\right]$) according to:

(2) $$\hat{y}=Tq$$

where the vector $\hat{y}$ collects the predicted values of the response while q are the regression coefficients expressed in terms of the scores (which are often called Y-loadings). Since the scores can be directly calculated from the predictor matrix through the weights:

(3) T=XR

($R=\left[r_{1}\;r_{2}\ldots \;r_{F}\right]$), the regression equation in (3) can be expressed explicitly in terms of the measured variables X as:

(4) $$\hat{y}=Tq=XRq=Xb$$

b=Rq being the regression coefficients.

#### 3.3.2. Sequential and Orthogonalized Partial Least Squares (SO-PLS)

Sequential and orthogonalized partial least squares [20,21] is a multi-block regression method conceived for modeling the relation between a response Y and a multi-set of predictors.

In the present study, two different instrumental platforms were used for data collection. This situation led to a multi-block data set constituted of two individual sets of independent measurements (X and Z), which can be jointly used for the estimation of the y response. This was achieved by a four-step algorithm, formulated as follows:

y is fitted to X by means of PLS;

Z is orthogonalized with respect to the X-scores, obtaining $Z_{orth}$;

Thus, the redundancies between X and Z that were modeled in Step 1 are removed.

The y-residuals resulting from Step 1 are fitted to $\;Z_{orth}$ by means of PLS

The final predictive model is estimated by combining the contributions of the two individual PLS regressions in Steps 1 and 3:

(5) $$\hat{y}=Xb+Z_{orth}c$$

where b and c are the regression coefficients associated to Step 1 and Step 3, respectively. If needed, and for easier interpretation, the global model in Equation (5) can be reformulated such that regression coefficients can link $\hat{y}$ directly to Z rather than to $\;Z_{orth}$.

## 4. Conclusions

The present work aimed to develop a green and rapid multi-platform tool for quantifying l-DOPA excess in DOPA racemic mixtures. In order to achieve this goal, MIR and NIR spectroscopies were used in combination with two different regression models: PLS and SO-PLS. The outcome of the chemometric analysis has highlighted that the quantification of l-DOPA in racemic mixtures is possible by coupling NIR and PLS, and, to a lesser extent, by using MIR. The multi-platform approach provided a higher accuracy than the individual block analysis, indicating the association of MIR and NIR spectral data by means of SO-PLS, which represents a valid solution for the quantification of the L-DOPA excess in racemic mixtures. Eventually, VIP analysis was used to understand which variables contribute the most to the solution of the regression problem. This further investigation made apparent that, from the MIR side, NH bonds of the NH_2_ and the C=O stretching of the carboxyl group was the greatest contribution to the model, whereas the most informative NIR variables were those related to carboxylic compounds and to humidity.

## Figures and Tables

**Table 1:** PLS analysis: tested pretreatment, number of LVs extracted and RMSECV.

**MIR Data—Calibration Models**			
**Model**	**Preprocessing**	**LVs**	**RMSECV**
Model Ia	Raw data (+MC)	13	18.8
Model IIa	First derivative (+MC)	8	18.3
Model IIIa	Second derivative (+MC)	9	24.3
Model IVa	SNV (+MC)	9	23.4
Model Va	SNV + First derivative (+MC)	9	18.1
Model VIa	SNV + Second derivative (+MC)	8	18.3
**NIR Data—Calibration Models**			
**Model**	**Preprocessing**	**LVs**	**RMSECV**
Model Ib	Raw data (+MC)	4	32.2
Model IIb	First derivative (+MC)	3	25.5
Model IIIb	Second derivative (+MC)	6	11.5
Model IVb	SNV (+MC)	3	27.7
Model Vb	SNV+ First derivative (+MC)	3	23.6
Model VIb	SNV+ Second derivative (+MC)	6	10.8

**Table 2:** Mass (g) of racemic DOPA and L-DOPA in mixtures.

N. of Sample	Sample Name	Mass of Racemic DOPA (g)	Mass of l-DOPA (g)	Total Sample Mass (g)	Enantiomeric Excess (%)
1	Dopa 000	0.60567	0.00000	0.60567	0.00
2	Dopa 001	0.61403	0.00627	0.62030	1.01
3	Dopa 003	0.58243	0.01818	0.60061	3.03
4	Dopa 005	0.57182	0.03014	0.60196	5.01
5	Dopa 007	0.55921	0.04213	0.60134	7.01
6	Dopa 010	0.54491	0.06062	0.60553	10.01
7	Dopa 012	0.52834	0.07202	0.60036	12.00
8	Dopa 015	0.51493	0.09050	0.60543	14.95
9	Dopa 020	0.48431	0.12165	0.60596	20.07
10	Dopa 025	0.45100	0.15118	0.60218	25.10
11	Dopa 028	0.43340	0.16803	0.60143	27.94
12	Dopa 030	0.42269	0.18258	0.60527	30.16
13	Dopa 035	0.39116	0.21281	0.60397	35.23
14	Dopa 037	0.38150	0.22040	0.60190	36.62
15	Dopa 040	0.35759	0.24661	0.60420	40.81
16	Dopa 045	0.33336	0.27181	0.60517	44.99
17	Dopa 050	0.30024	0.30083	0.60107	50.05
18	Dopa 055	0.26983	0.33322	0.60305	55.25
19	Dopa 060	0.24147	0.36331	0.60478	60.07
20	Dopa 063	0.22171	0.37875	0.60046	63.08
21	Dopa 065	0.21067	0.39487	0.60554	65.21
22	Dopa 070	0.18019	0.42144	0.60163	70.05
23	Dopa 072	0.16962	0.43234	0.60196	71.82
24	Dopa 075	0.15354	0.44841	0.60195	74.49
25	Dopa 080	0.12132	0.48260	0.60392	79.91
26	Dopa 085	0.09083	0.51198	0.60281	84.93
27	Dopa 088	0.07411	0.52830	0.60241	87.70
28	Dopa 090	0.06008	0.54253	0.60261	90.03
29	Dopa 093	0.04357	0.55858	0.60215	92.76
30	Dopa 095	0.02961	0.57519	0.60480	95.10
31	Dopa 097	0.02194	0.58238	0.60432	96.37
32	Dopa 099	0.00848	0.59434	0.60282	98.59
33	Dopa 100	0.00000	0.60565	0.60565	100.00

## Sample Availability

Samples of the compounds and/or corresponding spectra are available from the authors.
